# LncRNA HSPA7 in human atherosclerotic plaques sponges miR-223 and promotes the proinflammatory vascular smooth muscle cell transition

**DOI:** 10.1038/s12276-021-00706-8

**Published:** 2021-12-02

**Authors:** Soo-jin Ann, Hyoeun Bang, Chan Joo Lee, Jaewon Oh, Sungha Park, Seok-Min Kang, Jung Kyoon Choi, Sang-Hak Lee

**Affiliations:** 1 Integrative Research Center for Cerebrovascular and Cardiovascular Diseases, Yonsei University College of Medicine, Seoul, South Korea; 2 Department of Bio and Brain Engineering, KAIST, Daejeon, South Korea; 3 Division of Cardiology, Department of Internal Medicine, Yonsei University College of Medicine, Seoul, South Korea

**Keywords:** Mechanisms of disease, Atherosclerosis, Long non-coding RNAs

## Abstract

Although there are many genetic loci in noncoding regions associated with vascular disease, studies on long noncoding RNAs (lncRNAs) discovered from human plaques that affect atherosclerosis have been highly limited. We aimed to identify and functionally validate a lncRNA using human atherosclerotic plaques. Human aortic samples were obtained from patients who underwent aortic surgery, and tissues were classified according to atherosclerotic plaques. RNA was extracted and analyzed for differentially expressed lncRNAs in plaques. Human aortic smooth muscle cells (HASMCs) were stimulated with oxidized low-density lipoprotein (oxLDL) to evaluate the effect of the identified lncRNA on the inflammatory transition of the cells. Among 380 RNAs differentially expressed between the plaque and control tissues, lncRNA *HSPA7* was selected and confirmed to show upregulated expression upon oxLDL treatment. *HSPA7* knockdown inhibited the migration of HASMCs and the secretion and expression of IL-1β and IL-6; however, *HSPA7* knockdown recovered the oxLDL-induced reduction in the expression of contractile markers. Although miR-223 inhibition promoted the activity of Nf-κB and the secretion of inflammatory proteins such as IL-1β and IL-6, *HSPA7* knockdown diminished these effects. The effects of miR-223 inhibition and *HSPA7* knockdown were also found in THP-1 cell-derived macrophages. The impact of *HSPA7* on miR-223 was mediated in an AGO2-dependent manner. *HSPA7* is differentially increased in human atheroma and promotes the inflammatory transition of vascular smooth muscle cells by sponging miR-223. For the first time, this study elucidated the molecular mechanism of action of *HSPA7*, a lncRNA of previously unknown function, in humans.

## Atherosclerosis: Identifying noncoding RNA effect

A long non-coding RNA (lncRNA) called HSPA7 promotes the development of atherosclerosis, plaque in arteries. Many atherosclerosis-related genetic loci are in noncoding regions of genome, but there has been an incomplete understanding of them. Sang-Hak Lee at Yonsei University College of Medicine, Seoul, South Korea, and co-workers set out to identify a lncRNA involved in atherosclerosis and investigate its mode of action. Comparison of aortic tissues allowed them to identify lncRNAs more abundant in atherosclerotic tissue but less in healthy tissue. Of the 380 lncRNAs identified, only HSPA7 reliably increased when aortic cells were treated with a trigger of atherosclerosis. Inhibiting HSPA7 restored normal function in vascular cells, decreasing migration and inflammation. Further investigation showed that HSPA7 blocks the activity of miR-223, a microRNA that suppresses inflammation. These results identify a potential therapeutic target for atherosclerosis.

## Introduction

The risk of atherosclerotic cardiovascular diseases, such as coronary artery disease, is strongly attributable to genetic factors^1^. According to recent genetic studies, many novel genetic loci are associated with coronary artery disease but have an unknown function. In addition, many of them are reportedly located in noncoding regions of the human genome^2^. For example, the association between the 9p21 locus and myocardial infarction has been replicated by research groups. ANRIL, a noncoding RNA, was found to be located in this locus, and its function in influencing cell proliferation was partly elucidated^3^.

Long noncoding RNAs (lncRNAs) are the longest types of noncoding RNAs and are differentiated from other shorter noncoding RNAs. LncRNAs have very diverse functions and are regarded as attractive therapeutic targets due to their tissue, cell, and disease specificities^4^. As lncRNAs affect distal targets, they stabilize ribonucleoprotein complexes, alter phosphorylation pathways, or act as competing endogenous RNAs^5^. Dozens of lncRNAs have been reported to affect the development of atherosclerosis. Prior studies showed that specific lncRNAs regulate cells in arteries, including endothelial cells, vascular smooth muscle cells (VSMCs), and macrophages^4,6,7^. For instance, lncRNA H-19 is known to increase VSMC proliferation^8^, whereas lncRNA-p21 inhibits it^9^. Although some additional lncRNAs were identified to regulate vascular cells^10^, lncRNAs discovered from human vascular cells that affect atherosclerosis have been highly limited. Conversely, microRNAs inhibit target mRNAs usually via degradation or translational blocking^11^. Some microRNAs are involved in atherosclerosis by affecting SMC proliferation or phenotypic changes^12^.

In the current study, we aimed to analyze human atherosclerotic plaques and identify and functionally validate a lncRNA with a previously unknown function. We challenged VSMCs with several stimuli, including oxidized low-density lipoprotein (oxLDL), a hallmark of atherosclerosis, and evaluated the effects and mechanisms of action of the identified lncRNAs.

## Materials and methods

### Study subjects and aortic tissue extraction

The study protocol (no. 4-2013-0688) was approved by the Institutional Review Board of Severance Hospital, Seoul, Korea. All participants provided written informed consent. Aortic samples were obtained from patients who underwent aortic graft replacement surgery for a thoracic aortic aneurysm. Samples were classified by an experienced pathologist according to the presence of atherosclerotic plaques. The characteristics of the participants according to aortic atherosclerotic plaques are presented in Table 1. The pathologist classified the lesions according to the modified classification of the American Heart Association without any knowledge of the specimens^13^.

**Table 1:** Characteristics of study participants according to the presence of aortic atherosclerotic plaques.

	Atherosclerosis (*n* = 2)		Control (*n* = 4)			
Patient number	1	2	3	4	5	6
Age	79	72	68	48	61	73
Sex	Female	Male	Male	Male	Female	Female
Diabetes mellitus	No	No	No	No	No	No
Hypertension	Yes	Yes	No	Yes	No	Yes
Smoking	No	Yes	Yes	No	No	No
Hyperlipidemia*	No	Yes	No	No	No	No
Coronary artery disease	No	No	No	No	No	Yes
Body mass index	23.4	27.6	23.2	20.9	23.9	20.7

^*^Defined as low-density lipoprotein-cholesterol ≥160 mg/dL.

### RNA sequencing and analysis of lncRNAs

RNA was extracted from cells using a Ribospin RNA Extraction Kit (GeneAll, Seoul, Korea). RNA concentration and purity were assessed using a NanoDrop ND1000 spectrophotometer. Total RNA sequencing libraries were prepared using a TruSeq RNA sample preparation kit (Illumina, San Diego, CA, USA). The fragmentation step resulted in an RNA-Seq library that included inserts of approximately 100–400 bp. The average insert size in an Illumina TruSeq library was approximately 200 bp. cDNA fragments underwent an end repair process: the addition of a single ‘A’ base to the 3' end and then ligation of adapters. Finally, the products were purified and enriched with polymerase chain reaction (PCR) to create final double-stranded cDNA libraries. Libraries were quantified using KAPA Library Quantification kits for the Illumina HiSeq 2500 platform according to the protocol guide KK4855 (KAPA Biosystems, Wilmington, MA, USA). We normalized the RNA sequencing data by quantile normalization as previously described^14^. The differentially expressed genes between samples with and without atherosclerotic plaques were compared using Cuffdiff. Genes with upregulated and downregulated expression with *q*-values < 0.05 and >two-fold changes were identified.

### Cells and other reagents

Human aortic smooth muscle cells (HASMCs) were purchased from Lonza (Basel, Switzerland) and cultured in SMC growth basal medium containing growth factor supplemented with 2% fetal bovine serum and penicillin/streptomycin at 37 °C. THP-1 cells, a human monocyte cell line, were purchased from the Korean Cell Line Bank (Seoul, Korea). The cells were cultured in RPMI-1640 medium containing 10% fetal bovine serum and streptomycin (100 U/mL)-penicillin (100 μg/mL) at 37 °C with 5% CO_2_. THP-1 cells were plated in six-well dishes (1 × 10^6^ cells/well) and treated with 50 nM phorbol-12-myristate-13-acetate for 24 h for differentiation into macrophages. Lipopolysaccharide (LPS) and angiotensin II (AngII) were purchased from Sigma-Aldrich (St. Louis, MO, USA) for HASMC stimulation. Angiotensin II is a key peptide hormone in the renin-angiotensin-aldosterone system. This molecule exerts adverse cardiovascular effects by increasing vascular tone, fluid retention, thrombosis, and inflammation^15,16^. Low-density lipoprotein (LDL) for oxLDL was isolated from the plasma of healthy donors using sequential ultracentrifugation. LDL was dialyzed for 24 h at 4 °C with phosphate-buffered saline and oxidized for 24 h using 5 μM CuSO_4_ at 37 °C. Ethylenediaminetetraacetic acid was added to stop the reaction, and thiobarbituric acid reactive substance assays were used to analyze the oxidation state of LDL before each experiment.

### Migration assay

For analysis and comparison of HASMC migration, the cells were added to the upper Transwell chamber (Neuro Probe, Inc., Gaithersburg, MD, USA) in serum-free medium after transfection with si*HSPA7* or control siRNA for 24 h. The lower chamber was filled with SMC growth basal medium with fetal bovine serum. Next, LPS (10 ng/mL), oxLDL (50 μg/mL), or AngII (300 nM) was added to the upper chamber and incubated for another 24 h. Thereafter, the cells were stained with a Diff Quik staining kit (Kobe, Japan), and those on the lower surface of the filter were photographed and counted under a fluorescence microscope. All treatments were performed in duplicate wells. The sequences of si*HSPA7* are as follows: sense: UGAGCGUGACAGCCACUGACAGGAGCUU; antisense: GCUCCUGUCAGUGGCUGUCACGC UCAUU.

### Enzyme-linked immunosorbent assay (ELISA)

Cell culture supernatants were collected, and the amount of secreted IL-1β or IL-6 was quantified using ELISAs (R&D Systems, Minneapolis, MN, USA) according to the manufacturer’s protocol. Ninety-six-well plates were coated with 1 mg/well capture antibody. The coated plates were washed twice with phosphate-buffered saline containing 0.05% Tween-20. Following exposure to the medium, the assay plates were exposed sequentially to each of the biotin-conjugated secondary antibodies. The plates were read at an absorbance of 450 nm. Target proteins were analyzed according to the manufacturer’s specification. Appropriate specificity controls were included, and all samples were run in duplicate.

### Quantitative real-time PCR (RT-qPCR)

RNA was extracted from cells using a Ribospin RNA Extraction Kit (GeneAll, Seoul, Korea). The integrity of the extracted RNA was analyzed with a NanoDrop and quantified using spectrophotometric absorbance at 260 nm. Next, 1 µg of RNA was used for cDNA synthesis using an iScript™ cDNA Synthesis kit (Bio-Rad, Hercules, CA, USA). RT-qPCR was performed with a SYBR Green dye system on a LightCycler 480 real-time PCR machine (Roche, Basel, Switzerland) using a standard protocol. LightCycler software was used to analyze gene expression based on cycle threshold values normalized to β-actin expression. β-actin was used, as it is a commonly used housekeeper for normalization in RT-qPCR^17^. Amplified gene expression was assessed by melting curve analysis, and no reverse transcriptase or template controls were included. Analyses were performed in duplicates. RT-qPCR was used to verify lncRNAs detected by RNA sequencing and SMC phenotype marker genes. PCR primers are as follows. F: CATCCAGGTGTATGAGGTTGAG. R: CACGCTCAGGATGCCATTA. The *CNN1* and *TAGLN* genes encode calponin-1 and SM22α, respectively. These are markers of the contractile phenotype of SMCs. Inflammation is known to switch the vascular SMC phenotype from contractile to synthetic^18^.

### RNA-binding protein immunoprecipitation (RIP) assay

The RIP assay was conducted using an EZ-Magna RIP kit (Merck Millipore, Burlington, MA, USA) according to the manufacturer’s instructions. HASMCs were harvested and lysed in RIP lysis buffer. Cell extracts were then incubated with RIP buffer containing magnetic beads conjugated to anti-AGO2 antibody and IgG (Merck Millipore). Immunoprecipitated RNA was isolated, and qPCR analysis was performed to detect *HSPA7* and miR-223. The 2^−ΔΔCt^ method was used for quantification of RNAs, and U6 and β-actin were used as controls for miR-223 and *HSPA7*, respectively.

### Statistical analysis

All data are presented as the mean ± standard error of the mean. Analysis of variance, followed by Tukey’s test, was used to compare values of continuous variables between groups with post hoc analysis. Differences were considered statistically significant when the *p* value was <0.05. We used the software package Prism 5.0 for all data analyses (GraphPad Software, Inc., San Diego, CA, USA).

## Results

### Identification of lncRNAs associated with human atherosclerosis

**Fig. 1:**
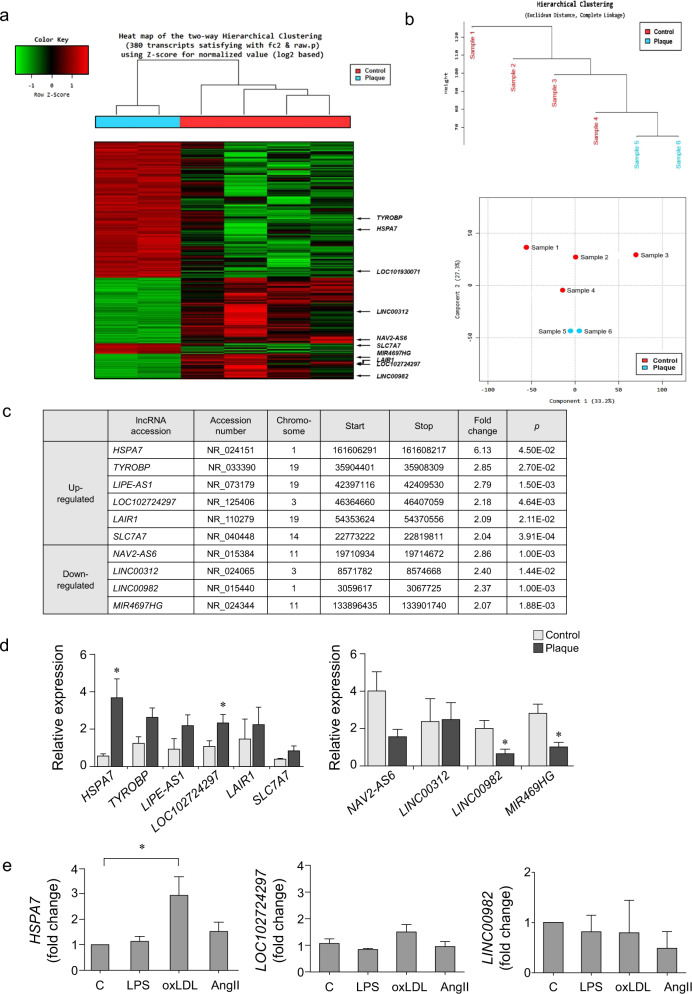
*HSPA7* expression is upregulated in human atherosclerotic plaques and induced by atherogenic stimuli. **a** Heatmap of genes differentially expressed in human atherosclerotic plaques compared to control tissues. **b** Hierarchical clustering and multidimensional scaling of data from plaques and controls. **c** List of high-ranking lncRNAs with up- or downregulated expression in plaques. **d** qPCR was performed to confirm lncRNAs with up- or downregulated expression using plaque and control tissues and four lncRNAs with significant differences. **e** Among the three top-ranked genes, only *HSPA7* showed a significant elevation upon proatherogenic stimuli, particularly oxLDL. Experiments were conducted in technical duplicates, and data are presented based on three independent replicates. *: *p* < 0.05. C control; oxLDL oxidized low-density lipoprotein.

RNA sequencing was performed to identify lncRNAs associated with atherosclerotic plaques. A total of 380 RNAs were found to be differentially expressed between plaques and controls (Fig. 1a). Hierarchical clustering and multidimensional scaling were conducted based on fragments per kilobase of transcripts per million mapped reads values (fold change > 2; *p* < 0.05), and the gene expression pattern in plaques was distinct from that of the controls. Multidimensional scaling visualized differences in gene expression between the two groups (Fig. 1b). Figure 1c presents high-ranking lncRNAs with up- or downregulated expression in plaques. qPCR to confirm the differential expression of lncRNAs showed four with significantly up- or downregulated expression. Among them, two lncRNAs with upregulated expression, *HSPA7* and *LOC102724297*, and one with downregulated expression, *LINC00982*, were selected for the validation experiment (Fig. 1d). When HASMCs were incubated with LPS, oxLDL, or AngII, only *HSPA7* showed upregulated expression, whereas the expression of the other two lncRNAs was not substantially changed (Fig. 1e).

### Knockdown of *HSPA7* attenuates migration and inflammatory changes in HASMCs

**Fig. 2:**
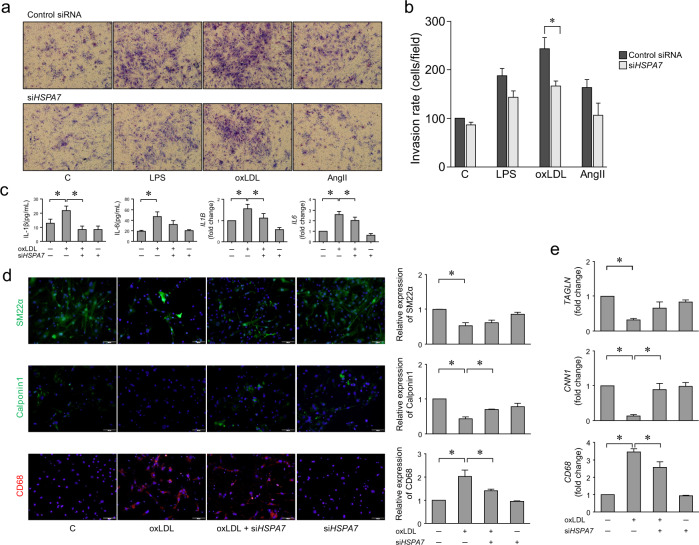
Knockdown of *HSPA7* attenuates migration and inflammatory changes in HASMCs. **a**, **b** Transwell assay for cell migration. HASMCs were transfected with si*HSPA7* or scrambled siRNA for 24 h and plated in the upper chamber with or without LPS, oxLDL, or AngII. The cells in the lower chamber were stained and counted. Knockdown of *HSPA7* significantly inhibited the oxLDL-induced migration of HASMCs. **c** ELISA and qPCR analyses showed that secretion and expression of IL-1β and IL-6 promoted upon oxLDL treatment were suppressed by si*HSPA7*. **d**, **e** Immunofluorescence staining revealed that SM22α and calponin1 were reduced upon oxLDL treatment, and this change was reversed by si*HSPA7*. The expression of CD68 upregulated by oxLDL was partly inhibited by si*HSPA7*. The effect of *HSPA7* on phenotype markers, particularly *CNN1* and *CD68*, was validated using qPCR. Data are from at least three independent experiments. *: *p* < 0.05. C control; LPS lipopolysaccharide; oxLDL oxidized low-density lipoprotein; AngII angiotensin II; HASMC human aortic smooth muscle cell; ELISA enzyme-linked immunosorbent assay; qPCR quantitative real-time polymerase chain reaction.

To examine the effect of *HSPA7* knockdown on HASMC migration, we transfected the cells with si*HSPA7* or control siRNA for 24 h and plated them in the upper Transwell chamber with or without LPS, oxLDL, or AngII. After another 24 h, analysis of the cells in the lower chamber revealed that migration of HASMCs promoted by oxLDL was significantly inhibited after si*HSPA7* treatment (Fig. 2a, b). After transfection with si*HSPA7* or control siRNA, HASMCs were treated with or without oxLDL for 24 h. ELISAs and qPCR showed that the oxLDL-promoted secretion and expression of IL-1β and IL-6 were suppressed by si*HSPA7*. The effect on IL-6 was not notable (Fig. 2c). Immunofluorescence staining showed that the expression of markers of the contractile SMC phenotype, SM22α, and calponin1, was decreased upon oxLDL treatment, and this change was reversed by si*HSPA7*. The expression of CD68, a marker of macrophage-like cells, was upregulated upon oxLDL treatment, whereas this change was partly inhibited by si*HSPA7* (Fig. 2d). The effects of *HSPA7* on phenotype markers, particularly *CNN1* and *CD68*, were validated using qPCR (Fig. 2e).

### *HSPA7* promotes inflammatory changes in HASMCs by sponging miR-223

**Fig. 3:**
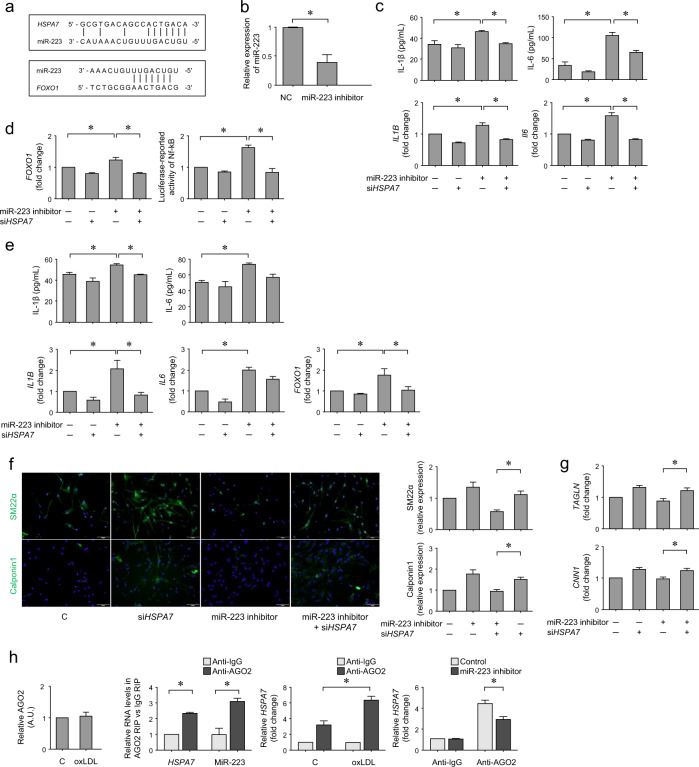
*HSPA7* promotes inflammatory changes in HASMCs by sponging miR-223. **a** Bioinformatics analysis using miRcode predicted that *HSPA7* contains a potential miR-223 binding site. miRDB revealed that FOXO1 was a target of miR-223. **b** After transfection of HASMCs with miR-223, qPCR showed significantly downregulated miR-223 expression. **c** HASMCs were transfected with a miR-223 inhibitor and/or si*HSPA7* or control siRNA and then treated with oxLDL. The secretion of IL-1β and IL-6 was increased upon miR-223 inhibitor treatment, whereas it was diminished after si*HSPA7* treatment, particularly for IL-6. These findings were verified using qPCR. **d** After the same treatment of HASMCs, qPCR showed that upregulated expression of *FOXO1* induced by the miR-223 inhibitor was diminished after si*HSPA7* treatment. Although luciferase-reported NF-κB activity was elevated upon miR-223 inhibition, this parameter was attenuated by si*HSPA7*. **e** After the same treatment of THP-1 cell-derived macrophages, the secretion of IL-1β and IL-6 was measured and showed a similar tendency to that in HASMCs. qPCR demonstrated up- and downregulated expression of the corresponding genes and *FOXO1*. **f** Immunofluorescence staining showed that the expression of markers of the contractile SMC phenotype did not change upon miR-223 inhibitor treatment; however, they increased after si*HSPA7* treatment. **g** These findings were verified using qPCR for *TAGLN* and *CNN1*. **h** miR-223 is a target of *HSPA7* in an AGO2-dependent manner. AGO2 expression was not different in HASMCs regardless of the presence of oxLDL. RIP assays showed that *HSPA7* and miR-223 were enriched in AGO2-containing miRNPs compared to IgG immunoprecipitates. *HSPA7* had increased binding to AGO2 in the presence of oxLDL. HASMCs were transfected with or without miR-223 inhibitor and then treated with oxLDL. *HAPA7* expression was shown to be significantly lower in AGO2 immunoprecipitates in the cells treated with the miR-223 inhibitor. Data are from at least three independent experiments. *: *p* < 0.05. HASMC human aortic smooth muscle cell; oxLDL oxidized low-density lipoprotein; C control; AGO argonaute-2; RIP RNA immunoprecipitation; miRNP miRNA ribonucleoprotein.

miRcode (http://www.mircode.org/) was used to search for candidate miRNAs interacting with *HSPA7*, and miR-223 had an optimal *HSPA7* binding site. First, 17 miRNAs were shown to bind *HSPA7*, and five miRNAs, miR-7/7ab, miR-96/507/1271, miR-182, miR-218/218a, and miR-223, that had higher conservation were selected. Among them, miR-182, miR-218/218a, and miR-223 were reported to affect IL-1β or IL-6 expression^19–21^. Finally, miR-223, which potentially affects the NLRP3 inflammasome^21^ and SMCs^22^ and has *HSPA7* binding sites not overlapping with other miRNAs, was selected as a candidate miRNA. miRDB (http://mirdb.org/) revealed FOXO1 as a target of miR-223 (Fig. 3a). In addition, FOXO1 has been reported to regulate Nf-κb^23^ and to influence smooth muscle cell proliferation^24^. Thus, we evaluated FOXO1 among targets of miR-223. HASMCs were transfected with a miR-223 inhibitor (Fig. 3b) and/or siHSPA7 or control siRNA and then treated with 50 μg/mL oxLDL for 24 h. IL-1β and IL-6 secretion increased upon miR-223 inhibitor treatment. The enhanced secretion of chemokines, particularly IL-6, was diminished with the addition of si*HSPA7*. These findings were verified using qPCR (Fig. 3c). After the same treatment of HASMCs, luciferase activity was measured after 48 h. qPCR showed that upregulated expression of *FOXO1*, a transcriptional activator of NF-κB, by the miR-223 inhibitor was diminished after si*HSPA7* treatment. Although luciferase-reported NF-κB activity was elevated upon miR-223 inhibition, this parameter was attenuated upon *HSPA7* knockdown (Fig. 3d). After the same treatment of THP-1 cell-derived macrophages, the secretion of IL-1β was increased with the miR-223 inhibitor, whereas it was decreased with si*HSPA7*. Although IL-6 exhibited a similar tendency to IL-1β, it was not significant. qPCR demonstrated up- and downregulation of the levels of *IL1B*, *IL6*, and *FOXO1* according to each treatment (Fig. 3e). In immunofluorescence staining, SM22α and calponin1 expression were not changed upon miR-223 inhibitor treatment, whereas the expression of these markers increased after the addition of si*HSPA7* (Fig. 3f). These findings were verified using qPCR, particularly that of *TAGLN* (Fig. 3g).

### *HSPA7* targets miR-223 in an AGO2-dependent manner

To evaluate whether *HSPA7* is associated with miRNA ribonucleoprotein complexes (miRNPs), we conducted an RIP assay in HASMCs using an AGO antibody. AGO2 expression was not different in the cells irrespective of the presence of oxLDL. RIP assays showed that *HSPA7* and miR-223 were enriched in the AGO2-containing miRNPs compared to the IgG immunoprecipitates. Furthermore, *HSPA7* binding to AGO2 was enhanced in the presence of oxLDL. To clarify the AGO2-dependent interaction between *HSPA7* and miR-223, we transfected HASMCs with or without miR-223 inhibitor and then treated them with oxLDL. In a RIP assay, *HSPA7* expression was significantly lower in the AGO2 immunoprecipitates in the cells treated with the miR-223 inhibitor (Fig. 3h).

## Discussion

Major findings of the current study include the following: (1) the expression of *HSPA7*, a lncRNA, was differentially upregulated in human atherosclerotic plaques and induced by oxLDL; (2) *HSPA7* knockdown inhibited the migration of HASMCs and secretion and expression of inflammatory mediators, whereas it recovered the expression of contractile markers of the cells reduced upon oxLDL treatment. (3) Although miR-223 inhibition promoted the activity or secretion of inflammatory proteins in HASMCs, *HSPA7* knockdown diminished these effects. (4) The impact of *HSPA7* on miR-223 was mediated in an AGO2-dependent manner. Notably, this study identified *HSPA7* in human atheroma and elucidated its function, although previous studies have not revealed the expression or function of *HSPA7* in vascular tissue. Our study not only reported the association of this lncRNA with atherosclerosis but also elucidated its biological effects on target vascular cells and the molecular mechanism of action. Furthermore, although the relationship between miR-223 and inflammation and vascular disease has been suggested, the current study first showed that miR-223 sponging is involved in the *HSPA7* pathway.

Several heat-inducible *HSP70* genes have been reported in humans, and *HSPA7*, also called *HSP70B*, is one of them. Similar to *HSPA6*, this gene is located on chromosome 1. Diverse expression levels of other *HSPA* genes have been shown in variable tissues, including blood, testis, and nerves^25^. Although *HSPA70B* mRNA is expressed in response to heat shock, this gene was found to encode no functional protein a decade ago^26^. Studies on this gene have been extremely limited since^27^. Here, for the first time, we identified the differential expression of *HSPA7* in human atheroma and validated its effect on VSMCs.

In our study, *HSPA7* affected VSMC status by sponging miR-223. miR-223 was reported to suppress the NLRP3 inflammasome in myeloid cells, and miR-223 dysregulation contributes to the pathogenesis of inflammatory diseases^28^. In addition, when miR-223 expression is upregulated in endothelial cells, inflammatory pathways are inhibited^21^. Furthermore, miR-223 knockdown in mice increased atherosclerotic lesions, which indicates the protective role of miR-223 in vascular disease^22^. A recent study conducted in patients with Kawasaki disease showed that miR-223 increases VSMC differentiation and that miR-223 deficiency causes VSMC dedifferentiation^29^. These findings also support that changes in miR-223 expression may be involved in the development of atherosclerotic vascular disease. Because we demonstrated that miR-223 was a target of *HSPA7* in atherosclerotic lesions, miR-223 inhibition by *HSPA7* might contribute to the progression of atherosclerosis. Moreover, our findings on miR-223 and VSMCs suggested that miR-223 might be an appropriate target for cardiovascular prevention or a candidate disease marker. Most importantly, our study identified that *HSPA7* is a regulator of miR-223 in the atherogenic vascular environment. Although regulation of *FOXO1* by miR-223 that is affected by *HSPA7* was shown in our data, the relationship between miR-223 and FOXO1 was previously reported. In more than one cancer cell line, overexpression of miR-223 downregulated FOXO1 expression and suppressed tumor cell proliferation^30^.

Here, we additionally showed the molecular mechanism of how *HSPA7* exerts its effects. However, to date, detailed action mechanisms have been elucidated for just a few lncRNAs in VSMCs^4,5^. Lnc-Ang362 is regulated when VSMCs stimulated with angiotensin II act as hosts for the transcript of miR-221 and miR-222, and it is also involved in VSMC proliferation^31^. LncRNA-p21 reportedly inhibits VSMC proliferation and induces apoptosis, while its effect is associated with transcriptional activation of p53^9^. SMILR (smooth muscle-induced lncRNA enhances replication) also regulates VSMC proliferation with the acceleration of the cell cycle by interacting with CENPF (centromere protein F) mRNA^32^. In contrast, it is only incompletely understood how other lncRNAs affect VSMCs, such as *ANRIL*, *SENCR*, *HAS*-*AS1*, and *H19*^4,5^.

In previous studies, lncRNAs involved in atherosclerosis have been discovered in mouse cells, as in the case of Mexis^33^. In addition, they were identified via genetic analysis even though the studies were performed in humans, similar to the case of *ANRIL*^34^. Otherwise, these lncRNAs were found in nonplaque human cells, as in the case of *SENCR*^35^. Studies identifying lncRNAs in human atherosclerotic plaque have been extremely rare, except for one study by Hung et al. that found PELATON using human carotid plaques^36^. The strength of our study is that we discovered *HSPA7* in human plaques and elucidated the relevant biological function.

Our study has potential limitations. We validated that *HSPA7* promoted the inflammatory transition of VSMCs by sponging miR-223. However, we cannot conclude that sponging miR-223 is the only mechanism of action for *HSPA7*, and additional studies may help elucidate the mechanism further. LncRNAs are poorly conserved across species compared to microRNAs, and mice do not have an *HSPA7* homolog. Therefore, it was difficult to use a mouse model for the additional verification of the effect of *HSPA7*. However, as mentioned, its discovery in human plaque and simultaneous elucidation of its mechanism of action have made our results scientifically meaningful.

In conclusion, *HSPA7*, a lncRNA identified in human atherosclerotic plaques, plays a role in the inflammatory transition of VSMCs stimulated by oxLDL. This process is mediated by miR-223 sponging by *HSPA7*. Notably, our study newly identified a lncRNA of previously unknown function in human plaques and elucidated its molecular mechanism of action. The current study suggests that *HSPA7* can be a potential therapeutic target for atherosclerotic vascular disease.

## Data Availability

The data underlying this article are available on reasonable request to the corresponding author.
